# The Impact of Transfer-Related Ischemia on Free Flap Metabolism and Electrolyte Homeostasis—A New In Vivo Experimental Approach in Pigs

**DOI:** 10.3390/jcm12206625

**Published:** 2023-10-19

**Authors:** Daniel Stephan, Sebastian Blatt, Julian Riedel, Katja Mohnke, Robert Ruemmler, Alexander Ziebart, Bilal Al-Nawas, Peer W. Kämmerer, Daniel G. E. Thiem

**Affiliations:** 1 Department of Oral and Maxillofacial Surgery, Facial Plastic Surgery, University Medical Centre, Johannes Gutenberg University Mainz, Augustusplatz 2, 55131 Mainz, Germany; sebastian.blatt@unimedizin-mainz.de (S.B.); bilal.al-nawas@unimedizin-mainz.de (B.A.-N.); peer.kaemmerer@unimedizin-mainz.de (P.W.K.); daniel.thiem@unimedizin-mainz.de (D.G.E.T.); 2 Department of Anaesthesiology, University Medical Centre, Johannes Gutenberg University Mainz, Langenbeckstraße 1, 55131 Mainz, Germany; julian.riedel@unimedizin-mainz.de (J.R.); katja.mohnke@unimedizin-mainz.de (K.M.); robert.ruemmler@unimedizin-mainz.de (R.R.); alexander.ziebart@unimedizin-mainz.de (A.Z.)

**Keywords:** free flaps, free flap failure, electrolyte concentrations, ischemia, thrombosis

## Abstract

Free flap tissue transfer represents the gold standard for extensive defect reconstruction, although malperfusion due to thrombosis remains the leading risk factor for flap failure. Recent studies indicate an increased immune response and platelet activation in connection with pathologic coagulation. The underlying cellular and molecular mechanisms remain poorly understood, however. The presented study, therefore, aims to investigate if transfer-related ischemia alters intra-flap metabolism and electrolyte concentrations compared to central venous blood after free flap transfer in pigs to establish a novel experimental model. Free transfer of a myocutaneous gracilis flap to the axillary region was conducted in five juvenile male pigs. The flap artery was anastomosed to the axillary artery, and intra-flap venous blood was drained and transfused using a rubber-elastic fixed intravenous catheter. Blood gas analysis was performed to assess the effect of transfer time-induced ischemia on intra-flap electrolyte levels, acid–base balance, and hemoglobin concentrations compared to central venous blood. Time to flap reperfusion was 52 ± 10 min on average, resulting in a continuous pH drop (acidosis) in the flaps’ venous blood compared to the central venous system (*p* = 0.037). Potassium (*p* = 0.016), sodium (*p* = 0.003), and chloride (*p* = 0.007) concentrations were significantly increased, whereas bicarbonate (*p* = 0.016) and calcium (*p* = 0.008) significantly decreased within the flap. These observations demonstrate the induction of anaerobic glycolysis and electrolyte displacement resulting in acidosis and hence significant tissue damage already after a short ischemic period, thereby validating the novel animal model for investigating intra-flap metabolism and offering opportunities for exploring various (immuno-) thrombo-hemostatic issues in transplantation surgery.

## 1. Introduction

In head and neck surgery, free flap transfer is one of the most commonly used techniques for reconstructing significant tissue defects, representing the gold standard for aesthetic and functional reconstruction following ablative tumor surgery or trauma [1]. Although the success rate of free flaps is high at over 93%, flap loss due to malperfusion is a major complication with significant consequences for the patient [2]. Therefore, frequent flap monitoring is mandatory for the early detection of malperfusion, allowing timely intervention and flap salvage. Beyond clinical assessment, which is still considered the gold standard although proven to be highly subjective [3], hyperspectral imaging (HSI) has been established as a powerful monitoring tool for free flaps [4], allowing earlier detection of malperfusion than clinical monitoring [5]. Besides external compression due to hematoma [6] and vessel kinking, arterial or venous malperfusion mainly arises from thrombotic vessel occlusion [7,8]. Although timely identification remains crucial, pathological coagulation’s underlying mechanisms are poorly understood.

Furthermore, even after successful flap reperfusion, the unavoidable initial ischemic insult due to the flap transfer can still inflict significant tissue damage. The duration of blood supply interruption until sufficient blood flow reestablishment through the microanastomosis varies with flap type (e.g., approximately one hour for free forearm flaps compared to at least two hours for free scapula flaps due to necessary patient relocation) and hence determines the magnitude of tissue damage. Ischemic effects on tissue are already well understood [9]. In brief, oxygen deficiency leads to anaerobic glycolysis, lactate, and proton accumulation, resulting in cytoplasmatic pH drop [10]. As a result, compensatory mechanisms are activated, and protons are excreted in exchange for sodium ions through the H^+^/Na^+^ exchanger, resulting in sodium influx and cell swelling. Sodium ions are further exchanged for calcium ions via the plasmalemma Na^+^/Ca^2+^ exchanger. Simultaneously, ischemia depletes intracellular ATP levels, rendering ATPases like the Na^+^/K^+^ ATPase inactive and thereby ultimately resulting in the culmination of the activation of calcium-dependent proteases [11,12,13].

After the re-establishment of sufficient tissue perfusion, additional tissue damage, so-called reperfusion damage, might occur, which can result in exacerbation of cell death via the generation of reactive oxygen species (ROS), endothelial impairment, calcium overload, and (cellular) inflammatory response [11,14]. These mechanisms have yet to be investigated, particularly in free flap surgery.

Tolerance to ischemia varies among different tissues, with the brain being highly susceptible to damage due to reduced blood supply [15], while muscle exhibits a significantly greater ischemia resistance [16]. Although extensive research has explored muscle tissue’s reaction to the extent and duration of prolonged ischemia primarily by employing tourniquet methods [17,18], only limited data exist on the early stages of tissue damage. Considering the importance of achieving the shortest possible blood flow interruption during free flap transfer in head and neck surgery, elucidating these initial changes remains crucial to understand the influence of ischemia on intra-flap metabolism. 

The quantification of flap viability and ischemic effects has been investigated via the appliance of microdialysis, which has been further proven to be an effective tool in free flap monitoring. However, its utilization has been predominantly limited to assessing post-transplantation lactate and glucose levels for early identification of flap failure [19,20,21,22,23]. 

In the present study, we investigated the effect of transfer-related ischemia on intra-flap metabolism about early alterations. For this purpose, autologous myocutaneous gracilis free flap transfer was performed in male juvenile pigs, including anastomosis of the flap artery and venous catheterization for intra-flap venous blood sampling. Intra-flap electrolyte concentrations, lactate and bicarbonate levels, blood gases, and hemoglobin concentrations were analyzed via blood gas analysis immediately after reperfusion and compared to central venous blood samples. The simultaneous establishment of a novel experimental animal model facilitates further investigation of cellular and molecular mechanisms within free flaps, including analyzing hemostatic processes and immunothrombotic principles.

## 2. Materials and Methods

### 2.1. Animals

Five juvenile male pigs (sus scrofa domestica; weight: 31 +/− 1.9 kg; age: 10–12 weeks) were used for the described experiment after approval by the State and Institutional Animal Care Committee (Landesuntersuchungsamt Rheinland-Pfalz, Koblenz, Germany; project number 23 177-07/G 21-1-080). Due to the thrombotic occlusion of one venous catheter, blood samples could only be obtained from four pigs. The experimental protocols were in accordance with the rules set out in Directive 2010/63/EU of the European Parliament and the Council of Europe adopted on the 22 September 2010 on the protection of animals used for scientific purposes. The “PHS Policy on Humane Care and Use of Laboratory Animals” (NIH publication No. 15–8013, revised 2015; https://olaw.nih.gov/policies-laws/phs-policy.htm accessed on 15 May 2023) was always followed. The described establishment of the experimental protocol represented a sub-project within a research project investigating the effects of fluid management in hemorrhagic shock as well as the effects of hemorrhagic shock and different therapeutical strategies on free flap vitality. It did, therefore, not increase the number of animal experiments within this prospective randomized animal experiment. All experiments were conducted in the animal facility of the Department of Anaesthesiology, University Medical Centre Mainz, Langenbeckstraße 1, 55131 Mainz, Germany, under continuous supervision by qualified and experienced personnel.

### 2.2. Anesthesia and Instrumentation

Animals were starved 6 h before the experiment to minimize the risk of aspiration during intubation, but water was accessible ad libitum at all times. To reduce stress, animals remained in their familiar environment as long as possible. They were sedated with an intra-muscular (i.m.) injection of Azaperone (4 mg/kg) + Ketamine (4 mg/kg) into the neck or gluteal muscle. After being left undisturbed until successful sedation was confirmed, pigs were transported to the animal laboratory within the sedation period. The transport was supervised and accompanied by a veterinarian with continuous monitoring of peripheral oxygen saturation. Upon arrival, anesthesia, intubation, and instrumentation were performed as previously described [24]. In brief, general anesthesia was induced via intravenous (ear) injection of fentanyl (4 µg/kg), propofol (4 mg/kg) and atracurium (0.5 mg/kg) and sustained during the whole experiment via continuous infusion of propofol (8–12 mg/kg/h) and fentanyl (0.1–0.2 µg/kg/h). Then, endotracheal intubation was conducted using a standard endotracheal tube (6.0 mm) and volume-controlled ventilation (Evita 2, Draeger, Lübeck, Germany): positive end-expiratory pressure of 5 cmH_2_O; tidal volume of 8 mL/kg; fraction of inspired oxygen of 0.4; inspiration to expiration ratio of 1:2; variable respiration rate to achieve end-tidal CO_2_ < 6 kPa). Anesthetized animals received an abdominal peripheral venous catheter for drug administration, a pulse contour cardiac output system (PiCCO, Pulsion Medical Systems, Munich, Bavaria, Germany, right femoral artery) together with central catheterization of the left femoral vein and artery for thermodilution-related cold saline application and fluid resuscitation. Ultrasound guidance was used for vascular catheterization.

### 2.3. Surgical Preparation

**Figure 1:**
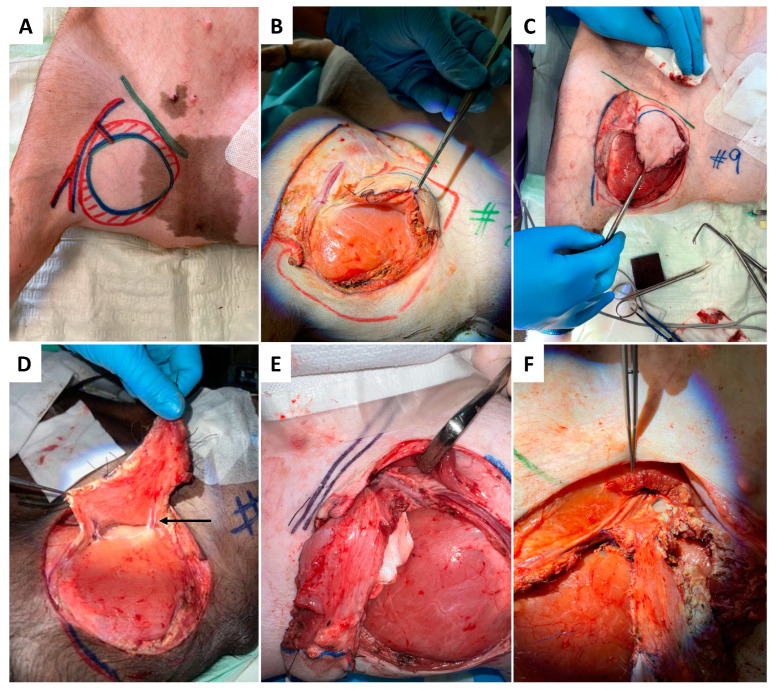
Anesthetized and intubated pig supine with abducted legs secured by bandages. Anatomical relevant structures were drawn in projection on the skin with color markers, including the inguinal ligament (green), the gracilis muscle (red-hatched), the skin paddle (blue ellipse), and the flaps’ vascular pedicle (**A**). The surgical procedure of flap harvesting begins with the skin incision at the inferior and medial border of the skin paddle (**A**), followed by subfascial flap preparation from medio-caudal towards later-cranial (**B**,**C**). The transaction of medial vessels (indicated by arrow) is presented in (**D**), followed by the dissection of the pedicle vessel at the superior and inferior (**E**,**F**) border.

After attaining a sufficient depth of anesthesia, animals were placed supine with the right upper and lower limp fully abducted and secured by bandages. Anatomical landmarks were drawn in projection on the skin, including external iliac and femoral vessels, gracilis muscle, skin paddle, and inguinal ligament (Figure 1A). Blood supply to the myocutaneous gracilis flap, which was first described by Finseth and Zimmerman 1979 [25], is provided by a thin artery originating from the external iliac artery (EIA) (Figure 1) [26,27,28], which arises from the abdominal aorta (AO) directly cranial to the inguinal ligament. Distally (6–7 cm) from the vascular bifurcation, EIA divides into the deep (DFA) and external/superficial femoral artery EFA, which further continuous as saphenous artery and vein (SA/SV) [29,30]. The saphenous artery is easily palpated at the inner thigh towards the proximal tibia and can be checked via ultrasound or Doppler device for confirmation. All surgeries were performed on the right half of the body with the donor site being ipsilateral to the recipient site. However, laterality of donor and recipient site should not influence the surgical procedure nor its outcome. The selection of the surgical site was due to technical and structural circumstances. The surgery required an essential surgical instrument set, a monopolar and bipolar caustic, and microsurgical equipment (KLS Martin, Gebrüder Martin GmbH & Co. KG, Tuttlingen, Germany). Surgeons used 3× optical magnification (StarMed GmbH & Co. KG, Grafing, Germany) for flap harvesting and 5.4× optical magnification for anastomotic suture.

### 2.4. Surgery

#### 2.4.1. Donor Site

**Figure 2:**
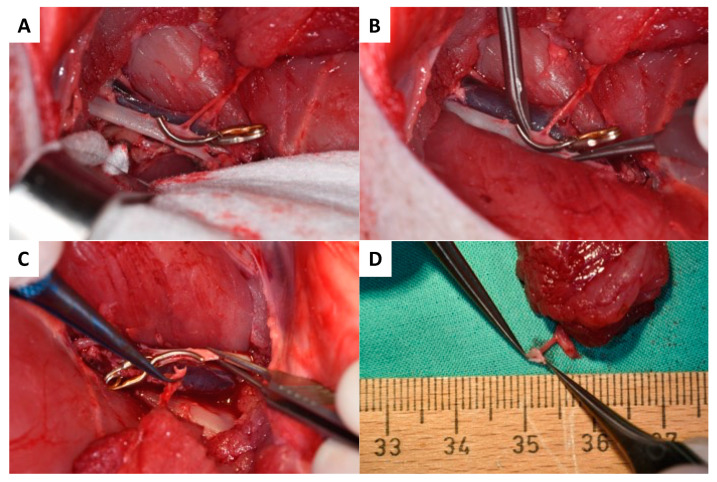
The surgical procedure of the EIA vessel set down begins with partial sealing (**A**) followed by partial dissection with a maintaining EIA vessel wall (**B**,**C**). The arterial pedicle vessel with the EIA vessel wall spindle is presented in (**D**).

**Figure 3:**
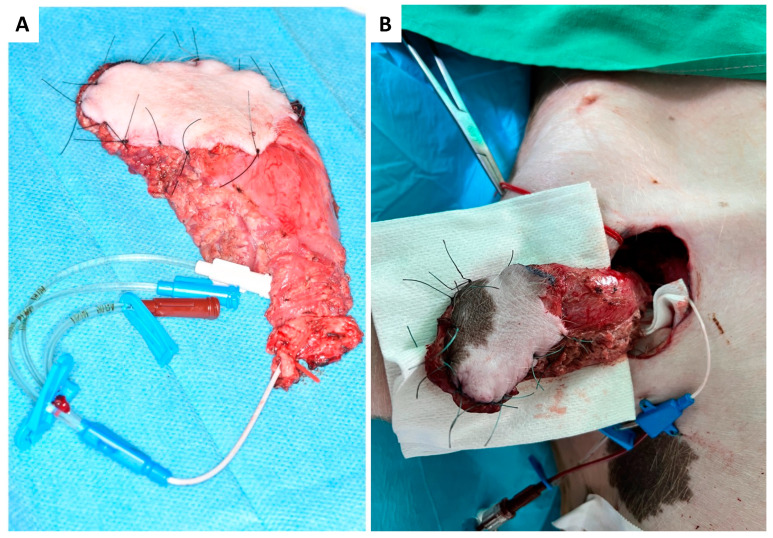
Harvested myocutaneous flap with catheterized venous pedicle and rubber elastic fixation (red vascular loop) before (**A**) and after arterial end-to-side anastomosis to the axillary artery and catheterization of the flap pedicle vein (**B**).

As presented in Figure 1A–F, skin incision was performed at the medial caudal border of the skin paddle along the inferior edge of the gracilis muscle (GM) with deep fascia suturing (Vicryl 3-0) to the skin paddle every 2 cm to avoid accidental shearing. Lower dissection was completed subfascial towards the saphenous vessels (SA/SV), which served as a lead structure to EIA/EIV. Although SV has been described as a vessel with variable occurrence [26], it was present in all five animals in our study. After distal ligation of SA and SV, the myocutaneous skin peddle was completely liberalized, including ligation of the medial nondominant neurovascular pedicle (Figure 1D). On average, the flap measured 10 cm × 8 cm. After identifying the central (lateral) vascular pedicle, the EIA and EIV were liberated to the inguinal ligament (Figure 1E,F). The flap remained perfused until the axillary recipient site was utterly exposed. EIA and EIV were partially sealed using a curved bulldog clamp, allowing the flap pedicle to set down while entraining an EIA vessel wall spindle to facilitate vessel anastomosis while maintaining limb perfusion (Figure 2A–D). Transection of the gracilis flap was followed by venous pedicle catheterization (LOGICATH^TM^ catheter, 4F, 3 lumens, ICU Medical, Inc., San Clemente, CA, USA) and fixation using rubber elastic vascular loops (Figure 3A). The flap was then flushed with 10–15 mL of heparin (500 mg/mL)/0.9% sodium chloride solution (1:5). 

#### 2.4.2. Recipient Site

**Figure 4:**
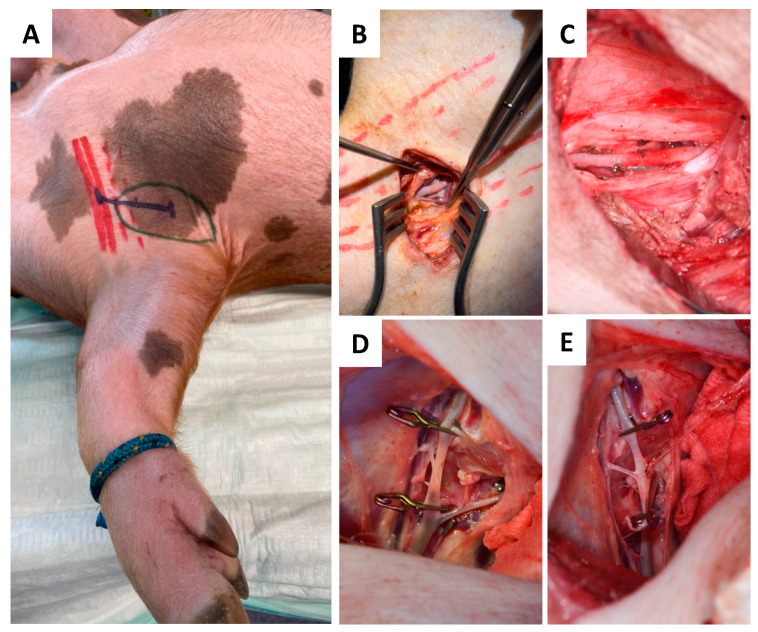
Schematic marks indicating the axilla (green), pectoralis muscle (red), and incision course (blue) (**A**) for dissection and skeletonization of the axillary artery (**B**). Liberalization of the axillary recipient artery (**C**) was followed by end-to-side micro anastomosis (**D,E**).

Due to its accessibility and the vessel diameter, the axilla was chosen as superior recipient site. To minimize surgery time, both flap raising and preparation of the axillary recipient site/vessels were performed simultaneously in a two-team approach. As shown in Figure 4, the skin incision was made perpendicular to the muscle fiber direction of the pectoral major muscle, followed by its partial transection to achieve access to the axilla. The axillary artery was liberalized by about 4–5 cm in length (Figure 4A–C).

#### 2.4.3. Anastomosis

At the recipient site, micro-anastomosis of the artery was performed end-to-side. In contrast, venous blood was continuously drained via the venous catheter and collected in a commercially available blood transfusion bag containing citrate. Collected total blood volume was quantified throughout the whole experiment and re-transfused after thrombus filtration to avoid embolism (CompoFlow RCC^TM^, Fresenius Kabi Deutschland GmbH, Bad Homburg, Germany; Figure 3B and Figure 4D,E). 

### 2.5. Flap Monitoring and Tissue Sampling 

**Figure 5:**
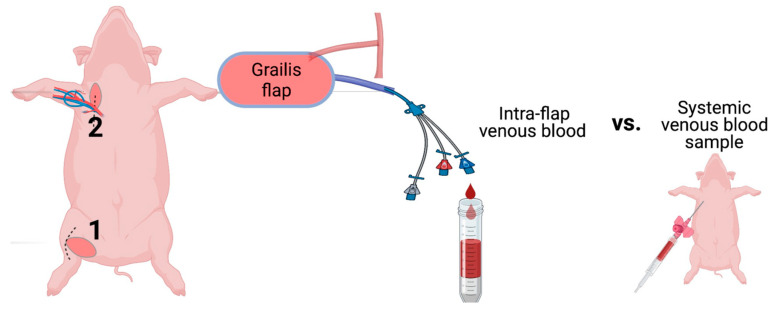
Schematic illustration of the experimental protocol. The gracilis free flap was harvested at the right inner thigh (1) and transferred to the axilla (2). The arterial anastomosis was conducted end-to-site to the axillary artery, whereas venous blood was drained for sampling and collected through catheterization of the pedicle vein. Central nervous blood samples served as a reference for blood gas analysis. (Figure created with BioRender.)

Blood samples (intra-flap venous blood and central venous blood) samples were collected immediately after flap reperfusion and used for blood gas analysis (ABL 90 Flex, Radiometer GmbH, Krefeld, Germany) assessing pH, metabolites, blood gases, electrolytes as well as hemoglobin types and concentrations. The experimental protocol is demonstrated schematically in Figure 5. 

### 2.6. Statistical Analysis

The software packages used for statistical analysis were GraphPad Prism 9.0 and Excel 16.76. Significance was set at *p* < 0.05. All data are presented as mean +/− standard deviation (SD). The effect of transfer time-induced ischemia on intra-flap acid–base balance, electrolyte concentrations, and hemoglobin types and concentrations compared to central-venous whole blood was analyzed using a 2-tailed Student’s *t*-test.

## 3. Results

The above-described experimental model was established to directly compare intra-flap and central venous blood samples to investigate intra-flap electrolyte levels, metabolism, and molecular mechanisms. 

### 3.1. Ischemia during Flap Transfer Changes the Intra-Flap Acid–Base Balance

**Figure 6:**
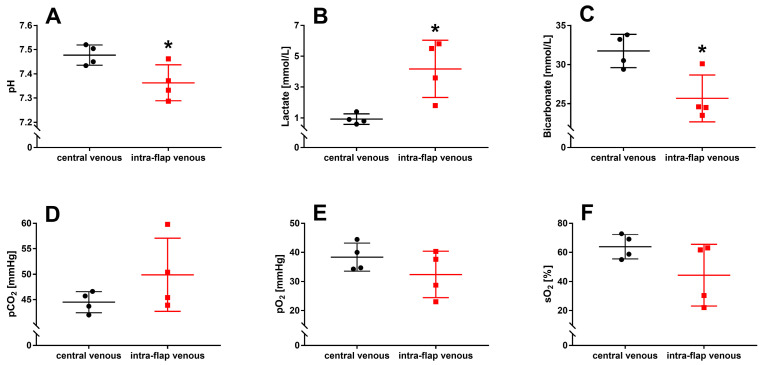
Intraoperative blood gas analysis with intra-flap (red) and central venous blood (black) were taken simultaneously following reperfusion. pH (**A**), concentrations of lactate (**B**), bicarbonate (**C**), partial pressures of carbon dioxide (pCO_2_) (**D**), oxygen (pO_2_) (**E**), and oxygen saturation (sO_2_) (**F**) were assessed via blood gas analysis. Data represent mean +/− standard deviation. Sample size: *n* = 4. * *p* < 0.05 and vs. central venous blood.

For blood gas analysis, a first intra-flap venous blood sample was collected after vascular anastomosis and reperfusion and compared with central venous blood. It revealed that ischemia during flap transfer leads to a significant decrease in pH (*p* = 0.037), an increase in lactate (*p* = 0.014), and a reduction in bicarbonate concentration (*p* = 0.016) within the flap (Figure 6A–C). No differences were found for partial carbon dioxide pressure (pCO_2_; *p* = 0.2), partial oxygen pressure (pO_2_; *p* = 0.2), and oxygen saturation (sO_2_; (*p* = 0.1) (Figure 6D–F).

### 3.2. Ischemia during Flap Transfer Alters Intra-Flap Electrolyte Concentrations

**Figure 7:**
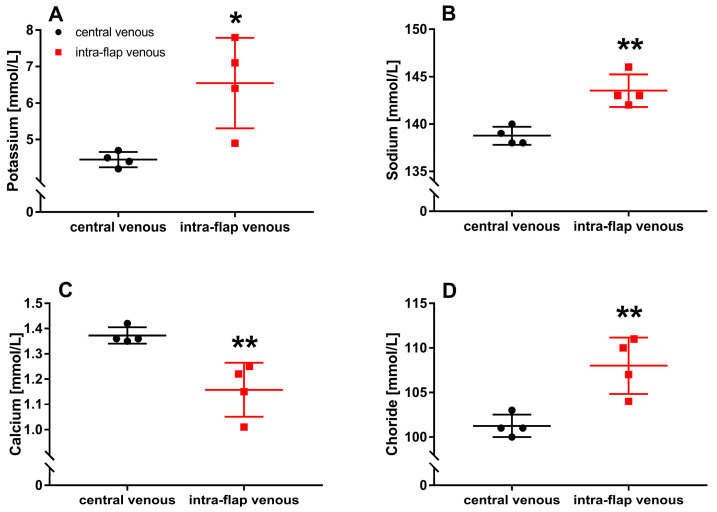
Intraoperative whole blood electrolyte concentrations. Intra-flap (red) and central venous blood samples (black) were taken simultaneously following reperfusion. Concentrations of potassium (**A**), sodium (**B**), calcium (**C**), and chloride (**D**) were assessed via blood gas analysis. Data represent mean +/− standard deviation. Sample size: *n* = 4. * *p* < 0.05 and ** *p* < 0.01 vs. central venous blood.

Ischemia during free flap transfer resulted in significantly increased potassium (*p* = 0.016), sodium (*p* = 0.003), and chloride (*p* = 0.007) concentrations within the flap compared with central venous blood samples. In comparison, the concentration of intra-flap calcium (*p* = 0.008) decreased significantly (Figure 7A–D).

### 3.3. Ischemia during Flap Transfer Has No Impact on Hemoglobin Types and Concentrations

**Figure 8:**
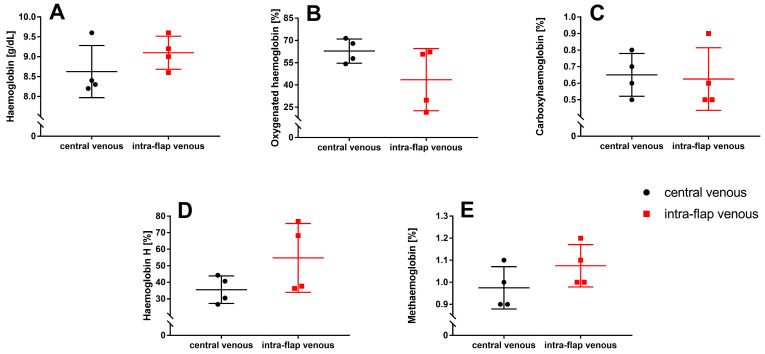
Intraoperative whole blood oximetry. Intra-flap (red) and central venous blood samples (black) were taken simultaneously following reperfusion. Hemoglobin concentration (**A**) and fractions of oxygenated hemoglobin (**B**), carboxyhemoglobin (**C**), hemoglobin H (**D**), and methemoglobin (**E**) were assessed via blood gas analysis. Data represent mean +/− standard deviation. Sample size: *n* = 4.

No difference between intra-flap venous blood and central venous blood could be observed regarding hemoglobin concentration (Hb; *p* = 0.3), oxygenated hemoglobin fraction (FO_2_Hb; *p* = 0.1), carboxyhemoglobin fraction (FCOHb; *p* = 0.8), hemoglobin H (FHHb; *p* = 0.1), and methemoglobin fraction (FMetHb; *p* = 0.2) (Figure 8A–E).

## 4. Discussion

To investigate the impact of transfer-related ischemia in free flap surgery on intra-flap electrolyte levels, metabolism, and hemoglobin concentrations in comparison to central nervous blood immediately after flap reperfusion, we established a novel porcine experimental set-up that, besides primates, provides the most excellent possible transferability to humans. Microvascular free tissue transfer was executed under identical conditions for head and neck reconstructive surgery. Upon flap harvesting, a catheter was placed in the venous pedicle to collect intra-flap venous blood samples. Immediately following reperfusion, venous blood gas analysis was performed on intra-flap and central venous blood, and the total tissue ischemia time during the tissue transfer averaged 52 ±10 min, leading to a discernible decline in pH (acidosis), increased lactate, and decreased bicarbonate levels within the flap. Serum electrolyte concentrations were further altered with elevated potassium, sodium, and chloride ions levels. They reduced blood levels of calcium compared to central nervous blood, all of which were statistically significant. Notably, ischemia did not exert any effects on hemoglobin types and concentrations.

Free flap transfer is the recognized standard in reconstructive surgery, predominantly utilized in head and neck procedures after tumor ablation surgery [1]. These procedures are notably extensive and complex, resulting in prolonged operative time, particularly in cases of primary reconstruction performed concomitantly with tumor resection. For these cases, operative time can range from five to ten hours, sometimes exceeding 20 h in challenging and demanding situations [31,32]. Operation time depends on multiple variables and influencing factors, including the case’s complexity, the surgical team and their experience, structural elements like operation environment and equipment, and previous treatments such as radiotherapy or previous operations. Despite optimizing surgical planning and efficiency, microvascular reconstruction remains an extensive procedure.

The association between extended surgical duration and adverse outcomes has been well investigated in various studies, establishing it as an independent and significant risk factor for flap failure and perioperative complication rates. For instance, Wong et al. noted a doubled risk of flap loss in patients whose surgeries reached or surpassed the 75th percentile in operative duration, equivalent to 625.5 min [32]. These findings are further supported by Irawati et al., with an incremental 11% increase in risk for postoperative complications for every additional hour of operation time [33]. Procedures extending a 10 h threshold represent a crucial risk factor for flap failure, thrombosis, bleeding, and hematoma [34]. However, a definitive cause-and-effect relationship cannot be established. While many previous studies have assessed the total duration of surgical procedures, none have differentiated between specific procedural steps and their respective characteristics. In flap ischemia, examining individual operative actions would be highly relevant since the extent of tissue damage has been proven to increase with a prolonged duration of blood flow interruption.

Tolerance to ischemia varies among tissues, with the brain being the most vulnerable organ to reductions in blood supply. Irreversible damage can manifest after 20 min of reduced organ perfusion [15]. On the contrary, muscle displays a significantly higher ischemia tolerance due to extensive carbohydrate storage and the ability to engage in limited anaerobic metabolism [16]. Belkin et al. observed a substantial muscle injury after an ischemia time of three hours (25% activity) with an aggravation of less than 3% functional activity after four, five, and six hours in a rat tourniquet ischemia model [17]. Concomitantly, a continuous increase in extracellular potassium concentration during three hours of ischemia with a rapid decrease upon tourniquet release has been demonstrated by Jennesche et al. [18]. While extensive tissue damage after prolonged ischemic conditions has been investigated extensively, only limited data regarding the early phase of tissue damage, particularly in muscle tissue, are available. Our findings indicate activation of anaerobic glycolysis in muscle tissue due to oxygen depletion after a short ischemic period, although proven to exert a high ischemia tolerance. 

This metabolic response is initiated by the previously well-described intracellular accumulation of protons and lactate, increasing excretion of metabolic products, concomitant with acidosis, electrolyte alterations, and serious cell injury [9,10,13]. Notably, the transfer-related duration of blood supply interruption needed to be more stable. Considering free scapular flaps with the necessity of patient repositioning, which significantly prolongs ischemia time, tissue damage can be suggested to be crucially increased in the clinical situation. Furthermore, the reperfusion phase following sufficient tissue transfer and blood flow reestablishment exerts additional risk to flap viability due to ROS generations, calcium overload, endothelial damage, and humoral and cellular immune response [10,14]. However, neither the tissue damage caused by ischemia nor the subsequent reperfusion injury in free flaps has been characterized in more detail, and therefore, further investigation is needed.

Microdialysis has been demonstrated as a functional method for examining tissue electrolyte concentrations within free flaps without possibly analyzing cellular blood components. In short, a physiological salt solution is slowly and constantly pumped through a semipermeable membrane, and the answer is equilibrated with the surrounding tissue fluid. Although representing a powerful tool for free flap monitoring [23], it exhibits certain limitations compared to our newly described model. Microdialysis predominantly has been used to quantify glucose, lactate, and pyruvate levels within the transplant, aligning with its primary focus on functional assessment of flap viability in the post-transplantation phase, aiming at early detection of perfusion disorders [19,20,21,22]. In contrast, our experimental model is designed to investigate intraoperative ischemia-related processes within the flap and subsequent reperfusion injury at molecular and cellular levels.

Our study’s small sample size (*n* = 4) poses challenges in generalizing results to a wider population, raising concerns about the influence of outliers or random variations. Despite observing significant mean differences, the substantial variability across data categories suggests limited result robustness. This variability, compounded by the small sample, can impact the precision of our estimates. Hence, our findings, though offering initial insights, should be approached cautiously. Future research should prioritize larger sample sizes to enhance statistical validity and provide a firmer foundation for our observations. In addition, no gene expression analyses nor immunhistochemical assessments for molecular validation were conducted in the presented study. The small sample size might reduce the validity. However, it aligns with ethical considerations while sufficiently proving the feasibility of the experimental model. The results are further in accordance with existing knowledge and well established principles of ischemia. Molecular analyses will be included in future studies.

In summary, the emergence of ischemia-induced acidosis and changes in electrolyte concentrations in the flap after a brief ischemic period during transfer underscores the clinical significance of minimizing ischemia duration to ensure successful tissue transfer. Simultaneously, the experimental model is validated, extending the utility of intra-flap venous blood samples to conduct molecular, immunohistochemical, and cellular analyses.

Outlook: The presented free flap animal model will be used in further experiments to investigate the underlying molecular and cellular mechanisms of pathological thrombus formation and coagulation within microvascular free flaps. For example, this experimental setup can investigate immunothrombogenesis due to surgery-induced ischemia formation or unavoidable endothelial damage. To the best of our knowledge, no animal or human studies are yet available exploring the relationship between hemostasis and immune response (immunothrombosis) by examining venous blood samples; hence, the presented experimental setup offers excellent potential for a deeper understanding. Concurrently, tissue samples (skin, muscle, vessels) can be readily acquired for mRNA and protein expression analyses, facilitating insight into the degree of tissue damage based on ischemia duration and severity. Flap perfusion can be further monitored using hyperspectral imaging as previously described by our group [4,5], evaluating tissue oxygen saturation (StO_2_), near-infrared perfusion index (NIP), as well as the tissue water (TWI) and hemoglobin index (THI). Controlled occlusion experiments of pedicle artery or vein could be performed to identify thresholds of critical hyperspectral perfusion parameters, respectively, to the time until occurrence after malperfusion. Controlled occlusion experiments can provide more detailed information on the dynamic of specific plasma-bound parameters and expression markers during malperfusion. Finally, with the described experimental protocol representing a sub-project within a research project investigating the effects of fluid management in hemorrhagic shock, the results of hemorrhagic shock and different therapeutical strategies on free flap vitality can be elucidated, too.

## 5. Conclusions

The presented results demonstrate significant tissue damage due to transfer-related blood flow interruption after a short ischemic period by activating anaerobic glycolysis, lactate accumulation and therefore acidosis followed by ion shifts and therefore cell death. To avoid irreversible tissue damage, ischemia duration should be kept as fast as possible. The feasibility of the newly designed animal model is further proved and enables its implementation in subsequent experiments aiming to understand molecular mechanisms of thrombus formation in free flaps.

## Data Availability

Data is contained within the article.
